# Enhancing provision of written medicine information in Australia: pharmacist, general practitioner and consumer perceptions of the barriers and facilitators

**DOI:** 10.1186/1472-6963-14-183

**Published:** 2014-04-23

**Authors:** Kim K Hamrosi, David K Raynor, Parisa Aslani

**Affiliations:** 1 Faculty of Pharmacy, The University of Sydney, Pharmacy & Bank Building A15, Sydney NSW 2006, Australia; 2 School of Healthcare, University of Leeds, Leeds LS2 9UT, UK

**Keywords:** Written medicine information, Patient education, Information-sharing, Barriers, Facilitators, Community pharmacists, General practitioners

## Abstract

**Background:**

Written medicine information can play an important role in educating consumers about their medicines. In Australia, standardised, comprehensive written information known as Consumer Medicine Information (CMI) is available for all prescription medicines. CMI is reportedly under-utilised by general practitioners (GPs) and community pharmacists in consultations, despite consumer desire for medicine information. This study aimed to determine consumers’, GPs’ and community pharmacists’ preferences for CMI provision and identify barriers and facilitators to its use.

**Method:**

Structured questionnaires were developed and administered to a national sample of Australian consumers (phone survey), community pharmacists and GPs (postal surveys) surrounding utilisation of CMI. Descriptive and comparative analyses were conducted.

**Results:**

Half of consumers surveyed wanted to receive CMI for their prescription medicine, with spoken information preferable to written medicine information for many consumers and healthcare professionals. GPs and pharmacists remained a preferred source of medicine information for consumers, although package inserts were appealing to many among all three cohorts. Overall pharmacists were the preferred provider of CMI primarily due to their medicine expertise, accessibility and perceived availability. GPs preferred CMI dissemination through both the GP and pharmacist. Some consumers preferred GPs as the provider of medicines information because of their knowledge of the patients’ medicines and/or medical history, regularity of seeing the patient and good relationship with the patient. Common barriers to CMI provision cited included: time constraints, CMI length and perceptions that patients are not interested in receiving CMI. Facilitators to enhance provision included: strategies to increase consumer awareness, longer consultation times and counseling appointments, and improvements to pharmacy software technology and workflow.

**Conclusion:**

Medicine information is important to consumers, whether as spoken, written or a combination of both. A tailored approach is needed to ascertain individual patient preference for delivery and scope of medicine information desired so that appropriate information is provided. The barriers of time and perceived attitudes of healthcare practitioners present challenges which may be overcome through changes to workplace practices, adoption of identified facilitators, and education about the positive benefits of CMI as a tool to engage and empower patients.

## Background

Written medicine information plays a pivotal role in educating consumers about medicines. Evidence suggests that written medicine information may influence patient knowledge and satisfaction [1], and have a beneficial effect on treatment adherence [2] and other health outcomes [3]. Written medicine information in the form of standardized Consumer Medicine Information (CMI) has been a topic of interest and debate since its introduction in Australia in 1993. Subsequent research [4,5], reports [6,7] and stakeholder consultations [8] have sought to gather information surrounding provision [6,7], utilisation [9,10] and impact [4,11]. Recently, the Therapeutic Goods Administration (TGA) sought public consultations on clarity around preparation, approval and maintenance of CMI, and its purpose and function [12].

CMI are standardised, brand-specific written information for consumers about prescription and pharmacist-only medicines prepared by pharmaceutical manufacturers [13]. CMI must be *available* to consumers either as a package insert or in a format enabling the information to be provided to the end-user of the medicine, for example electronically through healthcare professional dispensing or prescribing software, or via the Internet on Government and third-party websites [13]. The content must conform to regulations, but its *availability*, *format* and *provision* by healthcare professionals is not mandated [14]. Doctors and pharmacists however have a duty of care to provide medicine information, whether spoken and/or written, and as such guidelines have been produced to assist them in their legal and professional obligations [15-17].

Herxheimer suggested that written medicine information, such as CMI should satisfy three main criteria: firstly, what to expect from the medicine, secondly, how to use the medicine and lastly assist the consumer in communicating well with their healthcare professional about their medicine treatment [18]. Whilst CMI in Australia aims to address the first two criteria, evidence suggests CMI is under-utilised in counseling and receipt rates remain low [6,19]. Prior negative experiences, such as poor medication adherence after patients reading CMI, may influence perceptions of CMI. This may cause doctors and pharmacists to feel ambivalent about CMI provision and its use as a tool in educating consumers [9]. A recent exploratory study found time pressures during consultations, low patient confidence and communication skills, accessibility and insufficient demand for medicine information were barriers to patient CMI use [10]. Other studies have investigated pharmacists’ utilisation of CMI [7], including perceived obstacles to provision [5], however none have examined general practitioner (GP) practices.

An understanding of the barriers and facilitators, both perceived and actual, experienced by healthcare professionals and consumers in using CMI is necessary for the development of strategies and policies to promote increased use and better integration of CMI, and similar information resources, into daily practice. Therefore, informed by the findings of our two previous exploratory qualitative studies [9,10] on CMI use in Australia, we conducted a study which aimed to: (1) determine preferences for CMI provision and (2) identify the barriers and facilitators to the utilisation of CMI from the perspectives of consumers, GPs and community pharmacists.

## Methods

### Study design

Ethical approval was granted from the University of Sydney Human Research Ethics Committee to conduct this national study, which consisted of telephone surveys administered to consumers and mail surveys for healthcare professionals (GPs and pharmacists). The study was carried out between February and April 2009.

The consumer questionnaire was administered using a computer-assisted telephone interviewing system. Consumers were randomly selected from the Australian telephone directory and recruited using a pre-written script providing study information. Eligible consumers were: at least 18 years of age, able to participate without the need for a translator and taking at least one prescription medicine in the month prior to the survey. Responses were recorded directly into a database during the interview.

A random national sample (stratified by state using Australian Bureau of Statistics (ABS) [20] population data as the denominator and proportionally represented for metropolitan and rural populations) of 1100 community pharmacists and 1100 GPs was collected from a healthcare data information company database. The GPs and community pharmacists were invited to participate by a mailed postcard, prior to receiving the study pack containing study information and the questionnaire. A reminder and/or thank you postcard was sent two weeks after the initial survey. To increase response rates and encourage non-respondents, a further survey pack was sent four weeks after the initial survey.

### Sample size

Consumer sampling and recruitment was stratified by state and territory using ABS [21] population data to survey a representative sample based on age, gender and both metropolitan and rural populations. The standard error of proportions equation was used to calculate the sample size [22]. Although sample size calculations required a total of 226 consumers for the telephone survey, a sample size of 1000 consumers was used for comparative evaluation with a previous study [7].

Specifying a 5% degree of precision, and using a previously reported proportion of New South Wales (NSW) pharmacists providing CMI (7.6%) [5] the sample size for community pharmacists was calculated as 108. Assuming a 30% response rate, 360 pharmacists were required within NSW. The number of questionnaires sent to the remaining states and territories were stratified using the number of pharmacies per state/territory as the denominator, giving a total sample distribution of 1046, which was rounded up to 1100 subjects. There were no published data on the proportion of GPs or other prescribers providing CMI. Informed by response rates from other studies with medical practitioners (range: 47-68% [23,24]), a conservative 30% response rate was assumed and the GP sample size of 1100 subjects was selected to be consistent with the pharmacist sample.

### Questionnaires

The questionnaires were derived from earlier research [10] and previous findings [7,25]. A central questionnaire was developed and subsequently adapted specifically for each cohort: consumers, GPs and pharmacists. The structured questionnaires contained close-ended questions with single or multiple response options. The results in this paper are drawn from sections of the questionnaire focusing on two aspects:

•Future provision of CMI.

•Strategies to promote CMI awareness and use.

The questionnaires [6] and results derived from other sections have been published elsewhere [26].

The questionnaires were reviewed by a panel of pharmacists (n = 8), consumer representatives (n = 2) and experts in the field (n = 9) before piloting with four community pharmacists (postal questionnaire) and twenty-five consumers (telephone interviews). Based on feedback received, minor adjustments were made to the wording of the questions. Any changes derived from feedback were reflected across all three questionnaires.

All data were coded and entered into Statistical Package for Social Sciences (Version 19.0 IBM). Descriptive and frequency distributions were compiled and examined for each response before further analysis. Not all questions were answered and/or some allowed multiple responses hence the number of respondents varied for each question. Chi-square analyses comparing the barriers and facilitators and respondent demographic data were performed although no relevant statistically significant data were yielded.

## Results

### Participant demographics

The mail survey response rates were 34% (n = 349) and 17% (n = 181) for pharmacists and GPs, respectively. Researchers conducting the phone surveys called 11,653 telephone numbers nationally in both metropolitan and rural areas stratified according to ABS [21] demographic data to obtain the required 1000 eligible and consenting respondents. Potential participants were telephoned over a period of 21 consecutive nights between 6 pm and 8 pm local time, and up to five callbacks were made to establish contact. A total of 5386 persons answered the phone, of which 2107 people refused to participate with a further 1644 people not meeting the eligibility criteria, resulting in a response rate of 32%.

Females represented 52% (n = 516) of consumer respondents, with a median age of 60 (range 18–98) years and most spoke English as their first language (n = 970, 97%). Fifty-four percent (n = 189) of pharmacists were female with a median age of 47 (range 22–87) years, whilst 52% (n = 93) of GPs were female with a median age of 52 (range 31–83) years. The majority of pharmacists spoke English as their first language at home (n = 307, 88%), as did GPs (n = 158, 87%).

The occupations of consumer respondents consisted of: white-collar workers (n = 343, 34%); retirees (386, 39%); blue-collar workers (121, 12%); homemakers (85, 9%) and student/unemployed (47, 5%). Consumers’ education varied with 53% (n = 526) attaining a high school education, 37% (n = 370) a tertiary education, and the remaining a certificate or TAFE^a^ qualification. Pharmacists reported a median of 23 (Interquartile range (IQR) 7–33) years experience in their profession and primarily worked in independent (n = 185, 53%), or chain (n = 160, 46%) pharmacies (missing data n = 5). Approximately half (170, 49%) were owners or partners of the pharmacy, with the remainder permanent (140, 40%) or casual (29, 8%) employees. GPs had a median of 25 (IQR 16–31) years of experience and were in group (n = 152, 85%) or sole practices (n = 27, 15%).

### Preferred source and provider of CMI

Half (n = 500, 50%) of consumer respondents wanted to receive CMI for their prescription medicines. Consumers were asked to indicate two preferred sources of medicine information. Package inserts (n = 680, 68%) and computer-generated information from their doctor or pharmacist (n = 541, 54%) were reported as the two most preferred sources. A small proportion favoured information from a website (n = 126, 13%) or handwritten medicine information from their doctor or pharmacist (n = 98, 10%) and almost half (n = 435, 44%) only wanted spoken information from their doctor or pharmacist.

GP and pharmacist respondents ranked their preferred method of providing medicine information to their patients. The top three ranked sources by pharmacists (n = 349) were: CMI printed from the dispensing software (39%), spoken information (35%) and package inserts (23%). In comparison, GPs (n = 181) ranked spoken information (41%), package inserts (34%) and CMI printed from the prescribing software (15%) with some (14%) equally preferring written medicine information (other than CMI) from their prescribing software. Least preferred by pharmacists (1%) and GPs (1%) was to recommend a website.

Respondents were asked to nominate their preferred provider of CMI. Table 1 shows comparisons between GP, pharmacist and consumers responses. Both pharmacist (53.8%) and consumer (33.4%) cohorts saw CMI provision as the pharmacists’ role. In contrast, GP respondents felt the responsibility to provide CMI was *both* that of GP and pharmacist (46.4%). Few respondents wanted CMI accessed from the Internet.

**Table 1:** Consumer, pharmacist and GP preferences on CMI provider (role)

**CMI Provider**	**Consumer n = 500 (%)**	**Pharmacist n = 349 (%) (Missing n = 25)**	**GP n = 181 (%) (Missing n = 20)**
**Consumer: Who would you like to receive CMI from?**			
**Pharmacist/GP: Where do you think customers/patients should receive their CMI?**			
Pharmacist only	167 (33.4)	188 (53.8)	23 (12.7)
Doctor only	129 (25.8)	5 (1.4)	20 (11.1)
Both doctor and pharmacist	71 (14.2)	98 (28.1)	84 (46.4)
Neither doctor or pharmacist	12 (2.4)	NA	NA
Package insert	116 (23.2)	32 (9.2)	29 (16.0)
Internet website for CMI	3 (0.6)	0	1 (0.6)
Patients should not receive CMI	NA	1 (0.3)	4 (2.2)
Missing value	2 (0.4)	25 (7.2)	20 (11.0)
TOTAL	500 (100)	349 (100)	181 (100)

NA: Not asked.

The reasons for the allocation of role responsibility were explored (Table 2). Consumers considered both pharmacists and GPs as ‘experts in medicines’. They felt GPs were more aware of their medical history and medicines than pharmacists, however, easy access to and availability of pharmacists’ time made them attractive as a source of information, a view shared by both GPs and pharmacists. Both professions thought GPs as prescribers, should take more responsibility in providing CMI.

**Table 2:** Reasons for role responsibility of the pharmacist or doctor as most appropriate provider of CMI

**Why do you think this person (the pharmacist or GP as chosen) is the best person to provide CMI?**						
**Reason Given by respondents**	**Consumer Respondents**		**Pharmacist Respondents**		**GP Respondents**	
	**Pharmacist n = 167 (%)**	**GP n = 129 (%)**	**Pharmacist n = 188 (%)**	**GP n = 5 (%)**	**Pharmacist n = 23 (%)**	**GP n = 20 (%)**
The doctor/pharmacist is an expert on medicines	110 (65.9)	77 (59.7)	158 (84.0)	1 (20.0)	13 (56.5)	5 (25.0)
The doctor/pharmacist is aware of patient medical history and/or medicines	16 (9.6)	36 (27.9)	136 (72.3)	4 (80.0)	4 (17.4)	18 (90.0)
The doctor/pharmacist has more time to speak with the patient	19 (11.4)	1 (0.8)	84 (44.7)	1 (20.0)	9 (39.1)	1 (5.0)
The patient is able to access the doctor/pharmacist at any time	12 (7.2)	3 (2.3)	140 (74.5)	0 (0)	8 (34.8)	0 (0)
The doctor/pharmacist is prescribing/dispensing the medicine	11 (6.6)	9 (7.0)	104 (55.3)	4 (80.0)	12 (52.2)	14 (70.0)
The doctor/pharmacist sees the patient on a regular basis	8 (4.8)	9 (7.0)	133 (70.7)	0 (0)	1 (4.3)	10 (50.0)
The patient has a good relationship with the doctor/pharmacist	6 (3.6)	11 (8.5)	129 (68.6)	0 (0)	4 (17.4)	13 (65.0)
The patient is comfortable discussing their medicine with them	2 (1.2)	6 (4.7)	101 (53.7)	1 (20.0)	0 (0)	9 (45.0)
Other	14 (8.4)	2 (1.6)	0 (0)	0 (0)	0 (0)	1 (5.0)

Note: Responses are not mutually exclusive.

### Barriers to the provision of CMI

Responses were elicited from the three cohorts about the reasons for the non-provision of CMI in practice (barriers) by pharmacists and GPs. In the analysis these were subsequently categorised as either: situational (practice-related), financial (remuneration/cost of business), attitudinal (behaviours and perceptions) or cognitive (awareness, knowledge and understanding), based on previous research [27].

Table 3 shows the comparisons of the consumer, pharmacist and GP responses. Acknowledgement of time pressures was evident in the responses for all cohorts although this was more prominent for GPs. Financial barriers did not feature significantly in either cohort, except if CMI length was considered an issue for financial reasons (this distinction was not explored) that would relate to cost of time/labour and materials. The attitude of pharmacists/GPs featured prominently in consumer responses, most notably over a third of consumers reported that healthcare professionals think consumers only need to know what they are told, which makes the assumption that spoken information is sufficient for the consumer. Interestingly, the majority of pharmacists (61.3%) and almost half of GPs (42.8%) perceived that patients themselves are not interested in receiving CMI. The only cognitive barrier reported by pharmacists and GPs related to practice policy, and highlighted perhaps a lack of knowledge or awareness of professional obligations. This was more prevalent among GPs than pharmacists. Over a third of consumers reported CMI might not be provided to them because of healthcare professional concerns about their ability to understand the leaflet, which highlights consumers’ own awareness of issues of literacy and comprehensibility with health information.

**Table 3:** Barriers to the provision of CMI reported by GPs and pharmacists

**Barrier Type**	**Barriers to the provision of CMI**	**Consumers (%) n = 1000**	**Pharmacists (%) n = 349**	**GPs (%) n = 181**
	**What do you think are the reasons that you or other (pharmacists/GPs as appropriate) are unable to provide CMI to customers/patients?#**			
**Situational**	Limited time/too busy	527 (52.7)	196 (56.2)	141 (78.3)
	The CMI is not available in other languages	NA	116 (33.2)	41 (22.8)
	The CMI is too long to print off^*^	260 (26.0)	77 (22.1)	111 (61.7)
	CMI is difficult to provide because of the layout and workflow of the pharmacy	NA	27 (7.7)	NA
**Financial**	There is a lack of printers or printing costs are too high	NA	35 (10.0)	29 (16.1)
**Attitudinal**	Think consumers do not need to know more than what they are told by GP/Pharmacist	346 (34.6)	NA	NA
	Concern consumer may ask too many questions	291 (29.1)	NA	NA
	Patients are not interested in receiving CMI or GP/Pharmacist not interested in giving out	177 (17.7)	214 (61.3)	77 (42.8)
	The person is not a regular patient	224 (22.4)	32 (9.2)	22 (12.3)
	GP/Pharmacist concerned consumer won’t take the medicine	155 (15.5)	NA	NA
	I am always provided with a CMI	25 (2.5)	NA	NA
	Assume patient already knows about their medicine	18 (1.8)	NA	NA
	It is a repeat prescription	9 (0.9)	NA	NA
**Cognitive**	Concern information may be difficult to understand/read	345 (34.5)	NA	NA
	It is not the policy of the pharmacy or GP practice	NA	24 (6.9)	35 (19.4)
**Other**	No reason/don’t know	83 (8.3)	NA	NA
	Other	42 (4.2)	45 (12.9)	23 (12.8)

*Also considered as a financial barrier.

#Consumer questionnaire omitted “you or other” from the question.

NA: Not asked in questionnaire.

Note: Responses are not mutually exclusive.

### Facilitators to the provision of CMI

Respondents were also asked their thoughts on what would promote or facilitate CMI provision (Table 4). Consumer responses (40.4%) indicated that raising awareness of CMI as an available source of information about their medicines was a priority to increase CMI provision. This was reflected to a lesser extent in pharmacist (35%) and GP (26.7%) responses. Despite several consumer respondents indicating CMI provision should be made compulsory, this sentiment was not shared by either GP or pharmacist respondents. Issues of increasing available time or time saving measures were more prominent in pharmacist and GP responses. The majority of GPs and pharmacists indicated that having a summary (shortened version) CMI would facilitate provision. Over half of pharmacists and almost half of GPs wanted more time to provide and explain CMI, and around a quarter of both groups wanted counselling appointments to address provision. In contrast, consumers’ responses showed a decided lack of interest in the increased availability of healthcare professionals’ time to explain (8.2%) or specific appointments (0.9%) to discuss CMI, despite over half of consumer respondents previously acknowledging the time constraints experienced by healthcare professionals. In response to financial barriers, both pharmacists and GPs indicated increased reimbursement would facilitate CMI. Consumer respondents, however were less inclined to want to pay for CMI.

**Table 4:** Facilitators to the provision of CMI by GPs and pharmacists

**Barrier Type**	**Facilitators to the provision of CMI**	**Consumers (%) n = 1000**	**Pharmacists (%) n = 349**	**GPs (%) n = 181**
	**What do you think may help you or other pharmacists/GPs to provide CMI to customers/patients?#**			
**Situational**	More time to provide and explain CMI	82 (8.2)	184 (52.7)	81 (45.0)
	Having a self-serve computer in the pharmacy or surgery to print CMI	10 (1.0)	71 (20.3)	36 (20.0)
	Counselling appointments to discuss patients’ medicine	9 (0.9)	86 (24.6)	52 (28.9)
	Private area in a pharmacy to discuss the CMI	6 (0.6)	NA	NA
	Having CMI in different languages	6 (0.6)	114 (32.7)	52 (28.9)
	Dispensing software prompts to provide and record the provision of CMI	NA	164 (47.0)	78 (43.3)
	Tick box request by doctor on prescription form for pharmacist to provide CMI	NA	173 (49.6)	NA
**Financial**	If consumers paid for the CMI/increased reimbursement for provision of CMI	17 (1.7)	190 (54.4)	69 (38.3)
**Attitudinal**	Consumers having a regular pharmacist or doctor	23 (2.3)	NA	NA
**Cognitive**	Increased consumer awareness and request for CMI	404 (40.4)	122 (35.0)	48 (26.7)
	Legislate compulsory provision of CMI	143 (14.3)	0	0
	Having a summary CMI covering important points about the medicine	40 (4.0)	259 (74.2)	147 (81.7)
**Other**	Don’t know	145 (14.5)	0	0
	Other	91 (9.1)	6 (1.7)	6 (3.3)

#Consumer questionnaire omitted “you or other” from the question.

NA: Not asked.

Note: Responses are not mutually exclusive.

Finally, a note should be made regarding the chi-square analyses conducted comparing the barriers and facilitators and the respondent demographic data. No statistically significant findings were made in relation to GPs. Whilst there were some *statistically* significant associations relating to pharmacists’ gender, age and experience (e.g. gender (female) was associated with situational barrier of ‘CMI not in other languages’ χ2(1) = 5.389, p = 0.020; age (<47 years) and years of experience of pharmacists (<15 years) with the situational barrier of ‘lack of printers’ χ2(1) = 9.290, p = 0.001 and χ2(2) = 8.673, p = 0.013 respectively) these were not considered practically meaningful or relevant in the clinical or practice context.

## Discussion

This study identified consumers’, GPs’ and pharmacists’ preferences regarding the source and provider of medicine information, and determined the barriers and facilitators to the use of this information, in particular CMI.

The participating consumer sample was closely representative of the Australian population. In terms of gender the study contained 52% females, in line with the nominated sampling frame of 52.5% females. The median age for consumer participants was 60 years in comparison to 37 years for the Australian population [20] but this deviation could be explained by the specific targeting of medicine users whose higher median age was not unexpected as medication use and proportion of medicines used increases with age. Notably, the education levels of consumer respondents varied significantly, particularly the percentage of participants who held tertiary qualifications which was higher than ABS [28] reported data (37% vs 23%). This may be because those consumers with a higher level of education may have read medicine information in the past and were therefore more interested in participating in the study.

It is evident from the study findings that consumers want information about their medicines, each with their own differing needs and preferences. Healthcare professionals are still the preferred source of information for consumers, although a greater desire for package inserts in comparison to electronic CMI was found. This greater preference may be because consumers are guaranteed to receive the information without having to rely on healthcare professionals, or that they are likely to receive a CMI every time they collect their chronic medications and so do not need to retain previously received CMI.

Whilst the results indicated that half of consumer respondents wanted to receive CMI, about 44% only wanted spoken information about their medicine. Previous studies have indicated that patients view written medicine information as a back-up to spoken information [3] and are even resistant to receiving written information [29]. Education level may also play a role in determining preference for spoken information [30], as does patient trust in their healthcare professional [10]. All of these factors may explain why many consumer respondents only desired spoken information. Additionally, the findings may also reflect the preferences of the doctors and pharmacists, who favoured providing spoken information. Spoken information is easier to deliver especially where time is a barrier, and can be tailored to consumer needs. In addition, healthcare professionals may at times have (legitimate) concerns regarding consumers’ capacity to understand CMI and as such may elect to provide only spoken information. This may restrict the number of CMI being delivered, diminish consumer-healthcare professional interaction and limit consumer awareness of CMI. Whilst this may be justifiable (acknowledging CMI may not be the most appropriate information source for all consumers), the use of CMI as a counseling tool has many potential positive benefits that may result in enhanced patient familiarity and confidence with CMI, may increase its readership and use, and further empower patients with increased knowledge about their medicines. In the least, CMI should be an option offered at each consultation.

An important factor in determining where information is sought is access [31]. Pharmacists were preferred as a source of information over doctors. Pharmacists appeared to be perceived as ‘medicines experts’ by all cohorts, and were seen to be considerably more accessible to consumers and with time available (although pharmacists themselves did report time as a considerable barrier to provision). Pharmacists are frequently the last practitioner the patients may see before commencing treatment and their easy access and apparent ability to spend time with consumers without making an appointment makes them an attractive source of CMI. Further, professional obligations surrounding the provision of CMI, linked to remuneration contained within dispensing fees, may also explain why pharmacist respondents considered their profession as the preferred source of CMI.

In contrast, GPs predominantly saw *both* the doctor and pharmacist as having responsibility for providing CMI. GPs were selected by all three cohorts as a preferred provider of CMI predominantly because of their understanding and knowledge of patients’ medical history and/or medicines, regularity of seeing the patient, and their good relationship with the patient. The role of the doctor could be seen as ‘decision-maker’ due to being initiator of treatment and prescriber of medicines, and as such consumers want CMI at the time of prescribing or during consultation, as previously noted in the literature, for use in risk-benefit analysis, informed decision-making and for reassurance [4,10]. Of concern is the fact that only a small proportion of GP respondents saw themselves as a sole source of CMI which could mean that many consumers are missing out on receiving CMI, and although providing CMI to patients may not guarantee its use by consumers it may initiate a patient-professional dialogue about the medicine treatment.

Our results suggest patients may benefit from the clarification of role responsibility surrounding CMI provision. Informing patients about their medicine should be a collaborative and coordinated approach by both doctor and pharmacist determined within an inter-professional context to ensure comprehensive communication by all healthcare professionals during consultations and receipt of adequate information by patients about their medicines so that vital information is not missed. In addition, the understanding of healthcare professional roles in CMI provision may positively address reported time constraints and avoid gaps in information provision through diffusion of responsibility.

A surprise finding was how few respondents indicated a preference for the Internet as a source of written medicine information, considering the reportedly increasing use of the Internet to search for health and medicine information [32] and the increasing number of online CMI sources. Other studies have reported the Internet as a lesser source of information in comparison to doctors and pharmacists, and followed by written medicine information [33]. Although measures have been introduced in Australia to improve access to CMI through TGA and various third party provider websites, a lack of awareness by consumers of up-to-date, reliable and evidence-based Internet sources of information is evident. Issues of reliability and readability of information, and the ability to locate information sought on the Internet may be hampering its uptake by consumers [34,35].

The three major barriers to providing CMI to patients reported by GP and pharmacist respondents were limited time, the length of CMI and believing patients are not interested in receiving CMI. Pharmacists also expressed concerns regarding CMI not being available in other languages. Time constraints were also a barrier for consumers, however they reported healthcare professionals’ attitudes of consumers only needing to know what they are told and concerns surrounding comprehension of CMI as the main barriers to CMI delivery to consumers. In terms of facilitators, consumers considered increased awareness about CMI with a more proactive approach by consumers to request CMI as the most important facilitator. A second important facilitator for consumers was to legislate compulsory CMI provision (whether as a pack insert or by healthcare professionals was not determined). For GP and pharmacist respondents, strategies to facilitate CMI provision revolved around situational factors such as CMI in different languages, dispensing software prompts and in the case of pharmacists a means by which doctors could communicate their desire for CMI to be provided to the patient. Pharmacist and GP respondents did rank a summary CMI highly, although this was not reflected in consumer responses perhaps because consumer respondents believed this would mean less information. Lastly, the GP and pharmacist cohorts highlighted financial facilitators through increased remuneration, however consumers did not appear keen to pay for CMI.

As previously noted, the primary situational barrier to CMI provision was limited time, a point highlighted by all cohorts, which can have implications for the dissemination of CMI. Consultation length may influence patient understanding, satisfaction and desire for written medicine information [10,36]. Given sufficient time, practitioners have the opportunity to explore patient beliefs and preferences, and provide suitable written medicine information to their patients, and tailor the written information with verbal advice. In practical terms, increased consultation times may not be sufficient or possible, even though this was a significant situational facilitator reported in this study. It is virtually impossible given the current demands on practitioners to deal with chronic conditions and increasingly complex medicine regimes to firstly ascertain the appropriate amount of time for each patient’s situation and secondly spend the time necessary to discuss in detail the CMI content for each medicine. Notwithstanding, implementing strategies such as specific appointments to discuss patients’ medicines, broadening of paid medicine reviews, and delivery and reinforcement of information by all healthcare professionals involved in the care of a patient, may partly address the time burden on practitioners. Situational facilitators such as increasing efficiency to practice through upgrading of resources i.e. technical support and software, and changes to process to improve time management such as CMI workflow strategies, increased staffing, and in the case of pharmacists automated dispensing systems, may address and alleviate time pressures. The implementation of educational programs for healthcare professionals on the use of CMI in practice may also address the attitudinal barriers to CMI highlighted in this study and assist in its acceptance and adoption in everyday practice [5]. Furthermore, implementing the cognitive facilitators suggested in this study such as increasing consumer awareness through education campaigns and having a summary CMI as desired by pharmacists and GPs, may encourage CMI dissemination, foster the patient-professional dialogue and assist consumers to feel comfortable asking for CMI. Introducing a summary CMI may attend to the issues of CMI length, increase distribution and use by healthcare professionals, and effect consumer readership, as demonstrated in previous studies [37,38]. However, finding the balance between conciseness and detail may prove more difficult, with many studies indicating that patients, when given a choice, prefer expanded rather than brief information [3].

Although consumers also saw limited consultation time as a barrier, their perceptions of the attitudinal barriers affecting provision of CMI by doctors and pharmacists were noteworthy. Consumer respondents felt that doctors and pharmacists limited the amount of medicine information they provided to patients, which might be perceived as paternalistic or even withholding information. Whilst this may not be the intent of the practitioners in our study, limiting of information may be due to consideration of and concern for the psychological cost (anxiety, fear, worry) of patients reading about side effects and the potential for subsequent non-adherence [39]. Consumers also thought that doctors and pharmacists did not welcome questioning about CMI and their medicines. Many of these consumers may have wanted to engage in shared decision-making with their provider but felt the attitude of their provider precluded them from asking questions. Of recent years, there has been a change in the patient-provider relationship shifting from paternalism towards increasing patient autonomy. Doctors and pharmacists despite supporting patient autonomy may struggle with changing roles and the perceived undermining of their expertise and judgment as patients increasingly access medicine information and become involved in their treatment decisions [9].

Health care professionals may also underestimate consumers desire for information [40], which is consistent with our findings showing a large proportion of GP and particularly pharmacist respondents reporting consumers as not interested in receiving CMI, this was despite half of consumer respondents indicating they would like to receive CMI for their prescription medicines. Patients have a right to information and using CMI as a tool may foster useful discussion, encourage patient engagement with their treatment decisions and potentially improve their health literacy.

In interpreting the study findings, the following limitations should be considered. There may be bias towards participants with a specific interest in CMI, and the results have been derived from self-report data, and subject to personal, social desirability and/or recall bias. A representative consumer sample was achieved with regard to gender and location in accordance with ABS data, however, there was a greater proportion of participants with tertiary qualifications, and as would be expected, a higher proportion of older consumers. Details of the consumer participants’ current medications and/or medical conditions were not elicited and their responses may have been influenced by the seriousness or chronic nature of their treatment. The telephone surveys despite the advantages of rapid data collection and accessibility to respondents have certain limitations [41]. Inattentiveness, time constraints and survey length may have a negative affect on participant responses. Calls were limited to unrestricted landlines, and consumers with mobile telephones only or silent numbers may not have been represented. Additionally, respondents may have used call screen technology to avoid telemarketing calls and as such did not respond to the telephone call. The telephone survey response rate was unable to be compared to the previous telephone survey about CMI conducted by Benton *et al*. [7] as no response rates were provided. However, the study response rate is within the range of similar public telephone surveys in Australia which yielded response rates of 18.2% [42] and 47.9% [43] even when mobile phones [42,43], unlimited call cycles, and weekend/evening call times [42] were included. Disappointingly, GP response rates were lower than expected, despite follow-up, which may reflect the low priority that CMI has for invitees or time pressures experienced. Although the GPs’ and pharmacists’ respondent sample was not generalisable, the results may provide fresh insight into the use and provision practices of GPs and pharmacists in relation to CMI, providing a basis from which to direct further research.

## Conclusion

Doctors and pharmacists are still a preferred source of medicine information for consumers, despite the rapid expansion and increasing availability of CMI on the Internet. It is evident consumers want to receive information about their medicines and, whilst this study found healthcare professionals and many consumers often prefer spoken information, one in two consumers still want to be provided with CMI. Discussions with consumers surrounding the provision of information should be encouraged so that doctors and pharmacists may ascertain consumer preference for either spoken, written or a combination of both types of information and tailor this according to consumer needs. At the minimum consumers should receive spoken information about their medicines as low literacy or education may preclude them from effectively using CMI.

Whilst concerns regarding length of CMI, and time limitations as barriers to provision of CMI are valid, improvements to the CMI quality and length, and implementing systems into practice that encourage CMI provision may be of benefit. Addressing time limitations may be more challenging due to the increasing burden on healthcare professionals to manage increasingly complex and chronic conditions within limited consultation times, however targeted questioning, a collaborative inter-professional environment surrounding role responsibility and the implementation of healthcare professional education and/or on using CMI as a tool during consultations may ensure consumers are at the minimum being offered CMI and/or provided with direction to where CMI may be accessed at a later point should consumer preference for this information change. The findings pertaining to the barriers and facilitators offer an opportunity for improvements to workflow and time management in practice, and should be considered as the basis for future recommendations.

## Endnote

^a^Technical and Further Education (TAFE) is vocational education and training offering nationally recognized qualifications including Certificates, Diplomas, Advanced Diplomas, Graduate Certificates and Graduate Diplomas.

## Competing interest

D.K. Raynor is co-founder and academic advisor of Luto Research Ltd, which develops, refines and tests health information.

## Authors’ contribution

KH developed the survey instrument, conducted the study, performed the statistical analyses and wrote the manuscript. DR conceived the study, participated in its design and critically reviewed the paper. PA conceived the study and design, developed the survey instrument, assisted in conducting the study and critically reviewed the paper. All authors have read and approved the final manuscript.

## Pre-publication history

The pre-publication history for this paper can be accessed here:

http://www.biomedcentral.com/1472-6963/14/183/prepub
